# A nomogram-based optimized Radscore for preoperative prediction of lymph node metastasis in patients with cervical cancer after neoadjuvant chemotherapy

**DOI:** 10.3389/fonc.2023.1117339

**Published:** 2023-08-15

**Authors:** Conghui Ai, Lan Zhang, Wei Ding, Suixing Zhong, Zhenhui Li, Miaomiao Li, Huimei Zhang, Lan Zhang, Lei Zhang, Hongyan Hu

**Affiliations:** ^1^ Department of Radiology, The Third Affiliated Hospital of Kunming Medical University, Kunming, Yunnan, China; ^2^ Department of Radiation Oncology, The Third Affiliated Hospital of Kunming Medical University (Yunnan Cancer Hospital, Yunnan Cancer Center), Kunming, China; ^3^ 920th Hospital of Joint Logistics Support Force, Kunming, Yunnan, China; ^4^ Department of Radiology, The First Affiliated Hospital of Henan University of Chinese Medicine, Zhengzhou, Henan, China; ^5^ Department of Gynecology, Yunnan Tumor Hospital & The Third Affiliated Hospital of Kunming Medical University, Kunming, Yunnan, China; ^6^ Department of Pathology, The Third Affiliated Hospital of Kunming Medical University (Yunnan Cancer Center), Kunming, China

**Keywords:** cervical cancer, neoadjuvant chemotherapy, multi-parameter MRI, lymph node metastasis, radiomics

## Abstract

**Purpose:**

To construct a superior single-sequence radiomics signature to assess lymphatic metastasis in patients with cervical cancer after neoadjuvant chemotherapy (NACT).

**Methods:**

The first half of the study was retrospectively conducted in our hospital between October 2012 and December 2021. Based on the history of NACT before surgery, all pathologies were divided into the NACT and surgery groups. The incidence rate of lymphatic metastasis in the two groups was determined based on the results of pathological examination following lymphadenectomy. Patients from the primary and secondary centers who received NACT were enrolled for radiomics analysis in the second half of the study. The patient cohorts from the primary center were randomly divided into training and test cohorts at a ratio of 7:3. All patients underwent magnetic resonance imaging after NACT. Segmentation was performed on T1-weighted imaging (T1WI), T2-weighted imaging, contrast-enhanced T1WI (CET1WI), and diffusion-weighted imaging.

**Results:**

The rate of lymphatic metastasis in the NACT group (33.2%) was significantly lower than that in the surgery group (58.7%, P=0.007). The area under the receiver operating characteristic curve values of Radscore_CET1WI for predicting lymph node metastasis and non-lymphatic metastasis were 0.800 and 0.797 in the training and test cohorts, respectively, exhibiting superior diagnostic performance. After combining the clinical variables, the tumor diameter on magnetic resonance imaging was incorporated into the Rad_clin model constructed using Radscore_CET1WI. The Hosmer–Lemeshow test of the Rad_clin model revealed no significant differences in the goodness of fit in the training (P=0.594) or test cohort (P=0.748).

**Conclusions:**

The Radscore provided by CET1WI may achieve a higher diagnostic performance in predicting lymph node metastasis. Superior performance was observed with the Rad_clin model.

## 1 Introduction

Cervical cancer is the fourth most common malignant tumor in women worldwide in terms of morbidity and mortality according to epidemiological surveys (1). The quality of medical care has improved globally; however, the prognosis of cervical cancer remains poor, with its 5-year survival rate varying greatly with different risk factors (51–95%) (2). Lymph node metastasis (LNM) is the main risk factor associated with poor prognosis in patients with cervical cancer. The five-year survival rate of patients with lymphatic metastasis is significantly lower than that of patients without lymphatic metastasis. Several studies have also reported that patients with cervical cancer metastasized to the lymph nodes undergoing radical surgery show significant decreases in survival rates (3–5).

The National Comprehensive Cancer Network (NCCN) guidelines recommend radical hysterectomy (RH) for patients with stage IB1-IIB cervical cancer. However, traditional RH can seriously affect endocrine and reproductive functions (5). Neoadjuvant chemotherapy (NACT) was proposed in the 1980s. The NCCN recommends that patients with FIGO stage IB2-IIB should undergo RH after NACT (3).

NACT combined with surgical treatment significantly improves progression-free survival (6). The postoperative pathological results of patients with cervical cancer, with no significant difference in FIGO staging, receiving NACT combined with surgery reveal a decrease in the LNM rate. Studies have also reported that the 5-year survival rate of patients receiving NACT combined with RH for cervical cancer is significantly higher than that of patients undergoing surgery alone (6, 7).

RH may be combined with NACT to reduce the positive rate of lymphatic metastasis, especially among patients with stage IB1-IIB cervical cancer, which may increase the 5-year survival rate (8). NACT reduces the risk of lymphatic metastasis; however, the occurrence of lymphatic metastasis remains significant for the prognosis of patients with cervical cancer receiving NACT (9).

Lymphatic metastasis is currently identified using clinical and pathological detection methods. However, non-invasive approaches such as multi-parameter magnetic resonance imaging (mpMRI) have also been proposed (10). mpMRI includes T2-weighted imaging (T2WI), contrast-enhanced T1-weighted imaging (CET1WI), and diffusion-weighted imaging (DWI). DWI and CET1WI can be used to evaluate tumor blood perfusion and changes in tissue physiology, while T2WI can provide anatomical information of the lesion and pelvis; therefore, mpMRI non-invasively assesses the size and morphological characteristics of tumor lesions. However, the sensitivity of mpMRI for assessing lymphatic metastasis is relatively low (10–12).

Radiomics has been widely used in the quantitative assessment of tumor heterogeneity, including pre-disease staging; assessment of the efficacy of radiotherapy and chemotherapy; and assessment of survival (8, 13, 14). Radiomics uses texture features in medical images to build machine-learning models that can predict or evaluate disease-related information. In current MRI radiomics research, controversies regarding sequence selection remain, and little attention has been paid to whether the lymphatic metastasis of cervical cancer in patients who received NACT should be assessed using a radiomics model.

Therefore, we attempted to construct a superior single-sequence radiomics model for assessing lymphatic metastasis in patients with cervical cancer after NACT in this study. We combined clinical parameters to construct a combined imaging and clinical model to predict whether patients with cervical cancer have lymphatic metastasis after NACT.

## 2 Materials and methods

### 2.1 Patients and lymph node ratio analysis

This multicenter research was approved by the Ethics Committee of our (primary center) and the Ethics Committee of our secondary center. The patients enrolled in the study were divided into two groups. The first part of the study consisted of retrospectively analyzing the difference in the lymph node ratio between patients who received NACT-RH and RH in our hospital between October 2012 and December 2021. The study included 1099 patients who underwent type C radical resection and pelvic lymphadenectomy only for cervical cancer in the primary center from October 2012 to December 2021. The diagnosis and staging criteria for all the patients were based on the 2019 cervical cancer FIGO staging criteria, and the staging of all the patients was determined by two or more senior gynecologists. The inclusion criteria for the first half of the study were as follows: [1] successful radical surgery, negative margins, and no residual tumor; [2] multimodal MR scans performed within 15 days before surgery and surgical treatment following the NACT MR scan in the NACT group; and [3] age ≥20 years. Exclusion criteria were as follows: [1] radiotherapy or cervical conization before surgery and [2] other malignant tumors or concurrent major chronic diseases.

The study excluded 696 patients; thus, 403 patients were finally included. The patients were divided into NACT (NACT-surgery, n=230) and surgery groups (only surgery, n=173) based on whether they had received NACT before surgery. The incidence rate of lymphatic metastasis in each group was calculated based on the number of pathologically confirmed LNM following lymphadenectomy.


$$LNR=\frac{number\;of\;lymph\;node\;metastasis}{The\;total\;number\;after\;lymphadenectomy}$$

### 2.2 Radiomics analysis after NACT

The radiomics analysis for assessing lymphatic metastasis in patients in the NACT group (n=230) included patients from the primary center in the first part of the study and those who received NACT before surgery from the secondary center. The inclusion criteria for the second half of the study were: [1] successful radical surgery, negative margins, and no residual tumor; [2] standard NACT before surgery; [3] multimodal MRI performed preoperatively and within 15 days before surgery and after NACT; and [4] regular follow-up for 3 years. Exclusion criteria were as follows: [1] prior radiotherapy or cervical coning prior to surgery; [2] incomplete clinical or pathological data; [3] presence of other malignancies or major chronic diseases during the study period; [4] image artifacts; [5] other autoimmune diseases; and [6] repeated entry into the bed during the scanning.

A total of 230 patients from the primary center (54 patients of LNM and 176 patients of non-LNM) and 56 patients from the secondary center (14 patients with LNM and 42 patients with non-LNM) were included in the second part of the study. The patients from the primary center were randomly divided into a training cohort (n=162) and a test set (n=68) at a ratio of 7:3, and the patients from the secondary center were included as the external validation cohort. The training cohort was used to construct the model and adjust the parameters during the 10-fold cross-validation. The test cohort was used to evaluate the generalization performance of the model, which does not involve the process of feature selection, feature standardization, and model construction. The external validation cohort was used to avoid overfitting and improve repeatability.

### 2.2.1 Neoadjuvant chemotherapy

**Figure 1:**
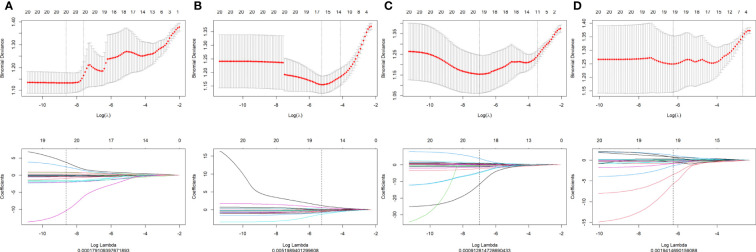
Texture reduction and selection through least absolute shrinkage and selection operator (LASSO) regression according to the minimum λ with 10-fold cross-validation. The Y-axis indicates binomial deviances, while the X-axis indicates the number of radiomics features based Λ **(A)** T1-weighted imaging (T1WI); **(B)** T2-weighted imaging (T2WI); **(C)** contrast-enhanced T1WI (CET1WI); **(D)** diffusion-weighted imaging (DWI).

**Figure 2:**
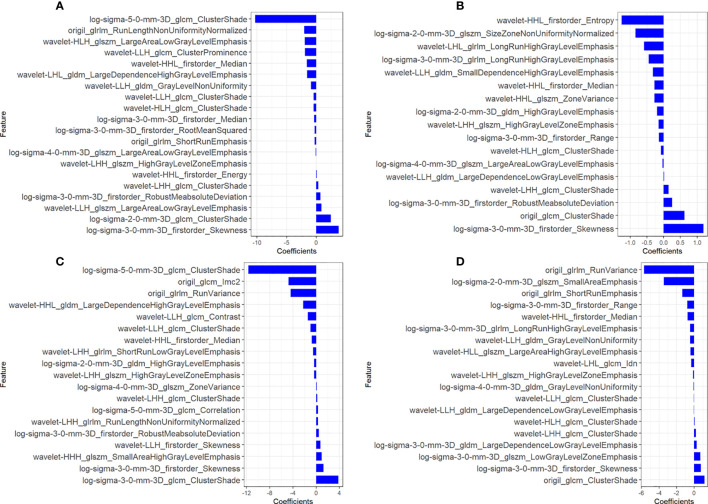
Coefficients of radiomics features used to construct the radiomics signatures of all four sequences. **(A)** T1-weighted imaging (T1WI); **(B)** T2-weighted imaging (T2WI); **(C)** contrast-enhanced T1WI (CET1WI); **(D)** diffusion-weighted imaging (DWI).

**Figure 3:**
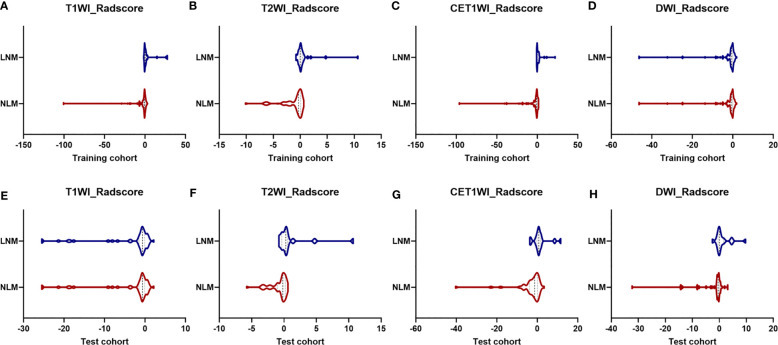
Differences in Radscore between patients with non-lymphatic metastasis (NLM) and lymphatic metastasis (LNM). The radiomics signatures of all four sequences differed significantly between patients with NLM and LNM (P<0.05) in the training cohort **(A)** T1-weighted imaging [T1WI]; **(B)** T2-weighted imaging [T2WI]; **(C)** contrast-enhanced T1WI [CET1WI]; and **(D)** diffusion-weighted imaging [DWI] and test cohort **(E)** T1WI; **(F)** T2WI; **(G)** CET1WI; and **(H)** DWI.

The indications for NACT were based on the 2019 FIGO guidelines for cervical cancer. According to the International 2019 FIGO staging standards, the cases were staged as massive IB2-IIA (maximum tumor diameter >4 cm) and IIB. All patients from the primary and secondary centers who had completed NACT and received the following medicine regimen were included: paclitaxel (175 mg/m^2^ D1) + cisplatin (75 mg/m^2^ D1); or paclitaxel (175 mg/m^2^ D1) + nedaplatin (80 mg/m^2^ D1). The interval between chemotherapy cycles was 3 weeks. NACT is generally performed for 1–3 cycles, and its efficacy is evaluated based on the objective response rate and adverse reactions (3). Before surgery, patients were evaluated for complete response (**Suppliment Material Figure 1**), partial response (**Suppliment Material Figure 2**), and progressive disease (**Suppliment Material Figure 3**).

### 2.2.2 Surgery

In the surgery group, 173 patients directly underwent surgery after completing examination, while the 230 patients in the NACT group underwent surgery 3–4 weeks after completing chemotherapy, which was completed by two senior associate chief physicians with more than 10 years of clinical experience. All patients underwent C-type radical cervical cancer surgery, bilateral pelvic lymph node resection, and para-aortic lymph node resection. The scope of lymph node resection included the internal iliac, external iliac, common iliac, obturator, presacral, and para-aortic lymph nodes (at the level of the inferior mesenteric artery).

### 2.3 Image acquisition and reconstruction of mpMRI

The MR images used in this study were those obtained before surgery and after NACT. Scanning at the primary center was performed with a supine head and an 8-channel body-part phased array abdominal coil, with the center of the coil facing the symphysis pubis on a 3.0-Tesla MRI machine (Inginia, Philips Healthcare, Netherlands). Before the examination, all patients were instructed to drink water sufficient to replenish the bladder appropriately, and 20 mg of scopolamine was injected intramuscularly 10 min before the scan to minimize intestinal peristaltic artifacts. MRI protocols included TIWI (repetition time [TR]/echo time [TE]: 546 ms/8 ms, field of view [FOV]: 300 × 340, slice thickness: 5 mm, slice gap: 1.5 mm, matrix: 332 × 332); T2WI (TR/TE: 3,881 ms/100 ms, FOV: 200 × 200, slice thickness: 4 mm, slice gap: 1.2 mm, matrix: 400× 297); CET1WI (TR/TE: 3.68 ms/0 ms, FOV: 300 × 340, slice thickness: 5 mm, slice gap: 0 mm, matrix: 280 × 138, enhancement contrast: Omniscan (GE healthcare, Ireland), 0.1 mmol/kg, 2 mL/s); and DWI (TR/TE: 6,000 ms/56 ms, FOV: 200 × 200, slice thickness: 4 mm, slice gap: 1.2 mm, matrix: 80 × 110, b value: 1200 s/mm).

Scanning at the secondary center was performed with a 16-channel body-part-phased array abdominal coil with the center of the coil facing the symphysis pubis on a 3.0-Tesla MRI machine (Discovery 750, GE Healthcare, USA). MRI protocols included TIWI (TR/TE: 850 ms/10 ms, FOV: 400 × 400, slice thickness: 5 mm, slice gap: 1.5 mm, matrix: 320 × 320); T2WI (TR/TE: 1300 ms/70 ms, FOV: 240 × 240, slice thickness: 4 mm, slice gap: 1.2 mm, matrix: 320 × 320); CET1WI (TR/TE: 10 ms/3 ms, FOV: 260 × 240, slice thickness: 5 mm, slice gap: 0 mm, matrix: 352 × 352, enhancement contrast: Omniscan (GE healthcare, Ireland), 0.1 mmol/kg, 2 mL/s); and DWI (TR/TE: 4,000 ms/80 ms, FOV: 400 × 400, slice thickness: 4 mm, slice gap: 1.2 mm, matrix: 160 × 120, b value: 1500 s/mm).

#### 2.3.1 Region-of-interest segmentation, image pre-processing, and feature extraction

The regions of interest (ROI) for the tumors were segmented on T1W images with the open-source software ITK-SNAP (version 3.6.0, www.itksnap.org). The most inferior and superior slices were excluded to minimize the effects of the partial volume. The two radiologists with 3–5 years of experience segmented the tumor ROI based on T1WI, following which the radiomics features were extracted and the inter-observer intraclass correlation coefficient (ICC) was calculated such that the features were maintained with an ICC inter value ≥0.7. Subsequently, a senior radiologist with over 10 years of diagnostic experience contoured the ROI twice. The senior physicians performed ROI segmentation according to the single-blind principle. After the first segmentation of the ROI, the ROI was segmented again after 6 weeks. The features were extracted from the results of the two ROIs, the ICC was calculated, and the parameters with ICC intra value ≥0.7 were retained. The features with ICC inter value ≥0.7 and ICC intra value ≥0.7 after the first segmentation were used for subsequent statistical analysis.

MATLAB 2016a (MathWorks, Natick, MA, USA) was used to reconstruct the images to provide the same spatial information (thickness, slice, and interlamellar space) to map the ROI for other single sequences of MRI. Before radiomics extraction, all images were co-registered, normalized (normalized scale: 100, as per the equation), and resampled to 1× mm^3^. All images were transformed into Gaussian and wavelets using Python (version 3.9.6) by the pyradiomics packages (github.com/Radiomics/pyradiomics). The radiomics features were extracted using Python by the pyradiomics package according to the feature guidelines of ImageJ (IBSI). Radiomics features were mainly extracted into six types: first-order features, shape features, gray-level co-occurrence matrix (GLCM), gray-level run-length matrix (GLRLM), gray-level size zone matrix (GLSZM), and gray-level dependence matrix (GLDM).


$$image\_standardization\frac{X-\mu }{adjusted\_stddev}$$

$$adjusted_{stddrev}=\;\max\;(\delta ,\frac{1.0}{\sqrt{N}}$$

#### 2.3.2 Radiomics signature construction

Four MRI sequences were used to construct the radiomics signature in this study. To optimize the research process of radiomics signature (Radscore) and reduce the waste of medical resources, we chose the optimal Radscore with the highest diagnostic performance among the four Radscores-based single-sequence radiomics signatures from patients from the primary center: Radscore_T1WI, Radscore_T2WI, Radscore_CET1WI, and Radscore_DWI. The optimal Radscore was selected based on the area under the receiver operating characteristic (ROC) curve (AUC) and the results of the DeLong test.

The datasets of the primary center were randomly divided into a training cohort and a test cohort at a ratio of 7:3. To predict LNM, the minimal redundancy maximum relevance (mRMR) algorithm, which can considerably improve the accuracy of feature selection and classification of the 30 features that had been kept, was used for initial feature selection in the training cohort. The least absolute shrinkage and selection operator (LASSO) method, which is suitable for the regression of high-dimensional data, was used to select significant distinguishable features based on minimum binomial deviance by adjusting the penalty coefficient (Λ) to construct the radiomics signature with 10-fold cross-validation.

#### 2.3.3 Clinical variables

The clinical variables included the clinical characteristics (age, sex, height, weight, body mass index [BMI], gestation number, parturition number); FIGO stage (IB1, IB2, IB3, IIA1, IIA2, IIB, IIC1p, IIIC2p) (15); neutrophil, lymphocyte, monocyte, platelet, hemoglobin, serum albumin, and tumor marker (squamous cell carcinoma-associated antigen [SCC-Ag], carcinoembryonic antigen [CEA], cancer antigen [CA]125) levels; human papillomavirus (HPV) levels; pathological type (15); lymph vascular space invasion (LVSI) (16); depth of stromal invasion; maximum tumor diameter on MRI; maximum tumor diameter determined *via* pathological examination, and histotypes (including squamous cell carcinoma, adenocarcinoma, and adenosquamous carcinoma) (17).

#### 2.3.4 Rad_clin model construction

The clinical variables from the training cohort were used to build the clinical and Rad_clin models to predict LNM. Clinical variables from the training cohort should be excluded with variance inflation factor ≥ at first. In the second, the clinical model was built using a multivariate logistic regression model based on the minimum Akaike information criterion (AIC). Subsequently, the Rad_clin model was built using a multivariate logistic regression model by clinical variable kept in the clinical model and the optimal Radscore, with 10-fold cross-validation to distinguish the status of LNM through a likelihood ratio test with backward step-down. A nomogram was constructed based on the Rad_clin model.

#### 2.3.5 Evaluation of model effectiveness

The AUC was used to predict the diagnostic performance of the models constructed using the training and test data and externally validated using the external validation data, and the Radscore of the patients in the test cohort and external validation cohort was calculated using the formula built-in the training cohort. The accuracy of the radiomics signature was evaluated for the training and test cohorts. The calibration of the models was assessed using calibration curves and the Hosmer–Lemeshow test. Decision curve analysis (DCA) was performed to estimate the clinical utility of the models.

### 2.4 Statistical analysis

Statistical analysis was performed using R 3.6.1 (www.Rproject.org). The R packages used in this study included tidyverse, caret, pROC, glmnet, DMWR, rmda, ggpubr, ModelGood, rms, mRMRe, DescTOOLs, and irr. Tenfold cross-validation was used at two points for recursive feature elimination to avoid random overestimation: [1] after the construction of the radiomics signatures by LASSO regression and [2] after the construction of the Rad_clin model. The DeLong test was used to compare the differences in the ROC curves between the two arbitrary models using Medcalc. The differences in the demographic and clinical variables were compared between the patients with non-lymphatic metastasis (NLM) and LNM in the training and test cohorts using GraphPad Prism 8 (www.graphpad-prism.cn). Based on the multiple comparisons being made, it was considered more appropriate to use a robust statistical test such as the Kruskal-Wallis nonparametric ANOVA and the Welch parametric ANOVA with *post hoc* correction for multiple comparisons. Chi-square tests were used to analyze categorical data.

## 3 Results

### 3.1 Incidence of LNM in the NACT and surgery groups

The NACT group consisted of 230 patients with cervical cancer, including 54 patients with LNM and 176 patients with NLM. A total of 173 patients were enrolled in the surgery group, including 64 with LNM and 109 patients with NLM. The average rate of lymphatic metastasis in the NACT group was 33.2%, which was significantly lower than that in the surgery group (58.7%, P=0.007).

### 3.2 Patient characteristics and clinical features

The demographic and clinical variables of patients in the training, test, and external validation cohorts are presented in **Tables 1**, **2**. No significant differences were observed in demographic data, including age, height, weight, BMI, gestation number, parturition number, laboratory test results, diameter by pathological, FIGO stage, diameter by MRI, depth of stromal invasion, and LVSI, between the training and test cohorts, indicating that patients were randomly divided into the training and test cohorts (P>0.05, **Table 1**). The lymphocyte (P=0.004, **Table 2**), blood platelet (P=0.001, **Table 2**), SCC_Ag (P=0.014, **Table 2**), and HPV (P=0.029, **Table 2**) levels were significantly lower for patients with NLM than for those with LNM in the training cohort; however, the difference was not significant in the test and external cohorts (**Table 2**). The tumor diameter on MRI was significantly smaller for patients with NLM (3.7 ± 1.3) than for those with LNM (4.2 ± 1.3) in the training cohort (P=0.039, **Table 2**). The same trend was observed in the test (P=0.011, **Table 2**) and external validation cohorts (P=0.044, **Table 2**). The FIGO stage was significantly different between patients in the NLM and LNM groups in the training (P<0.0001, **Table 2**) and test cohorts (P<0.0001, **Table 2**). The same trend was observed in the external validation cohort (P=0.016). Additionally, the tumor diameter based on pathological examination and depth of invasion showed significant differences between the patients with NLM and LNM in the external validation cohort (**Table 2**).

**Table 1:** Clinical information of patients in the training and test cohorts.

	Training cohort n=162	Test cohort n=68	P value
Age, years	47.1 ±8.8	46.8 ±9.0	0.820
Height, cm	159.0 ±6.4	158.4 ±6.2	0.507
Weight, kg	58.4 ±9.0	58.1 ±8.9	0.788
BMI	23.1 ±3.3	23.1 ±3.3	0.928
Gestation number	3.6 ±1.5	3.9 ±1.8	0.147
Parturition number	2.1 ±1.1	2.3 ±1.2	0.194
Laboratory values			
Neutrophils	4.2 ±1.7	4.1 ±1.9	0.754
Lymphocytes	1.9 ±1.6	1.9 ±0.8	0.886
Monocytes	0.3 ±0.1	0.3 ±0.2	0.203
Blood platelets	281.1 ±73.0	272.9 ±73.6	439
Hemoglobin	127.6 ±20.6	125.9 ±22.6	0.600
Serum albumin	44.6 ±4.1	44.8 ±5.2	0.756
SCC_Ag	7.4 ±18.9	4.8 ±6.3	0.122
CEA	11.4 ±55.9	13.6 ±61.8	0.801
HPV	563.1 ±718.1	413.1 ±596.7	0.102
Tumor diameter on MRI, cm	3.8 ±1.3	3.9 ±1.3	0.668
FIGO stage			0.403
IB1	19	12	
IB2	62	26	
IB3	11	4	
IIA1	17	8	
IIA2	13	4	
IIB	24	8	
IIIC1p	15	3	
IIIC2p	1	3	
Diameter pathology, cm	2.8 ±1.4	2.6 ±1.4	0.299
Depth of stromal invasion			0.952
0	7	2	
1	43	17	
2	52	23	
3	60	26	
Lymph vascular space invasion	5 (7.4%)	25 (15.4%)	0.148
Histotypes, n. (%)			0.49
Squamous cell carcinoma	140 (86.4)	59 (86.8)	
Adenocarcinoma	17 (10.5)	9 (13.2)	
Adenosquamous carcinoma	5 (3.1)	0 (0.0)	

BMI, body mass index; SCC_Ag, squamous cell carcinoma-associated antigen; CEA, carcinoembryonic antigen; HPV, human papillomavirus; MRI, magnetic resonance imaging; FIGO, International Federation of Gynaecology and Obstetrics; *P<0.05 indicates a significant difference.

**Table 2:** Clinical information of patients with non-lymphatic metastasis (NLM) and lymph node metastasis (LNM) in the training, test, and external validation cohorts.

	Training cohort		P value	Test cohort		P value	External validation cohort		P value
	NLM n=124	LNM n=38		NLM n=52	LNM n=16		NLM n=42	LNM n=14	
Age, years	47.1 ± 8.6	45.8 ± 10.3	0.426	47.5 ± 8.9	45.9 ± 8.5	0.523	47.3±9	47.8±3	0.890
Height, cm	158 ± 6.2	159.9 ± 6.2	0.089	158.5 ± 6.5	160.8 ± 6.1	0.203	159.2±5	161.6±0	0.297
Weight, kg	57.6 ± 8.6	59.8 ± 9.6	0.173	57.6 ± 8	61.2 ± 11.6	0.158	58.5 ± 3	63.0 ± 5	0.149
BMI	23.1 ± 3.2	23.4 ± 3.8	0.578	22.9 ± 3.1	23.6 ± 4	0.467	23.1 ± 4	24.1 ± 1	0.359
Gestation number	3.9 ± 1.8	4.1 ± 2.1	0.588	22.9 ± 3.1	23.6 ± 4	0.467	3.5 ± 3	4.4 ± 0	0.174
Parturition number	2.3 ± 1.1	2.3 ± 1.5	0.911	1.9 ± 1	2.6 ± 1.3	0.041*	2.2 ± 9	2.5 ± 3	0.395
Laboratory test									
Neutrophils	4.1 ± 1.8	4.1 ± 2.5	0.972	4.1 ± 1.8	4.3 ± 1.4	0.762	3.9 ± 1.8	3.9 ± 1.3	
Lymphocytes	1.8 ± 0.8	2.2 ± 0.6	0.004*	1.9 ± 0.6	1.9 ± 0.7	0.818	1.6 ± 0.5	2.1 ± 0.5	0.902
Monocytes	0.3 ± 0.2	0.3 ± 0.1	0.193	0.3 ± 0.1	0.3 ± 0.1	0.324	0.3 ± 0.1	0.3 ± 0.1	0.004*
Blood platelets	262.1 ± 67.6	307.9 ± 82.3	0.001*	272.3 ± 65.5	309.6 ± 89.8	0.069	264.4 ± 68.2	316.7 ± 88.7	0.644
Hemoglobin	127.7 ± 22.4	120.4 ± 22.4	0.081	128.7 ± 21.4	124.2 ± 17.9	0.455	130.1 ± 17.7	123.6 ± 21.3	0.067
Serum albumin	44.9 ± 5.1	44.7 ± 5.4	0.823	44.6 ± 4.4	44.6 ± 3.1	0.990	44.9 ± 5.9	45.2 ± 4.8	0.863
SCC_Ag	4.2 ± 5.4	7 ± 8.4	0.014*	7 ± 21.1	8.9 ± 9.6	0.720	2.7 ± 3.5	8.6 ± 9.9	0.055
CEA	14.8 ± 70	9.7 ± 17.3	0.659	12.3 ± 63.2	8.5 ± 19.2	0.815	13.8 ± 71.0	8.3 ± 19.8	0.660
HPV	357 ± 552.6	596.4 ± 699.3	0.029*	499.1 ± 664.5	771 ± 861.4	0.182	470.7 ± 607.1	848.9 ± 860.2	0.159
MRI findings									
Diameter, cm	3.7 ± 1.3	4.2 ± 1.3	0.039*	3.6 ± 1.3	4.4 ± 1	0.011*	3.2 ± 0.8	3.8 ± 0.8	0.044*
FIGO stage			<0.0001*			<0.0001*			0.016*
IB1	16	3		12	0		7	0	
IB2	54	8		22	4		24	6	
IB3	11	0		4	0		0	0	
IIA1	12	5		5	3		8	4	
IIA2	9	4		2	2		2	3	
IIB	22	2		7	1		0	1	
IIIC1p	0	15		0	3		0	0	
IIIC2p	0	1		0	3		0	0	
Pathological									
Diameter, cm	2.5 ± 1.4	2.8 ± 1.4	0.283	2.5 ± 1.3	3.6 ± 1.1	0.003*	2.9 ± 1.0	3.5 ± 0.9	0.071
Depth of invasion			0.890			0.027*			0.042*
0	6	1		2	0		0	0	
1	34	9		16	1		0	0	
2	39	13		19	4		26	8	
3	45	15		15	11		14	6	
LVSI	2	2	1.000	3	2	0.723	2	6	0.949
Histotypes, n. (%)			0.307			0.674			/
Squamous cell carcinoma	110 (88.7)	30 (78.9)		44 (84.6)	15 (93.8)		/	/	
Adenocarcinoma	10 (8.1)	7 (18.4)		8 (15.4)	1 (6.2)		/	/	
Adenosquamous carcinoma	4 (3.2)	1 (2.6)		0 (0.0)	0 (0.0)		/	/	

BMI, body mass index; SCC_Ag, squamous cell carcinoma-associated antigen; CEA, carcinoembryonic antigen; HPV, human papilloma virus; MRI, magnetic resonance imaging; FIGO, International Federation of Gynaecology and Obstetrics; LVSI, lymph vascular space invasion; DSI, depth of stromal invasion; *P<0.05, indicates a significant difference.

The parturition number was significantly smaller for patients with NLM (1.9 ± 1) than for those with LNM (2.6 ± 1.3) in the test cohort (P=0.041, **Table 2**). The monocytes showed significant differences between patients with NLM and those with LNM in the external validation cohort (P=0.004, **Table 2**) but not in the training and test cohorts. Other demographic data did not differ significantly between the NLM and LNM groups in the training, test, and external validation cohorts (P>0.05, **Table 2**).

### 3.3 Constructed radiomics signature

A total of 1,127 features (first-order statistics, shape-based texture, GLCM, GLRLM, GLSZM, GLDM, and neighboring gray tone difference matrix) per sequence were extracted from all four MRI sequences. After inter-observer ICC analysis, 943 radiomics features were retained (ICC>0.7). Four radiomics signatures (Radscore) were first constructed using single MRI sequences of T1WI, T2WI, CET1WI, and DWI separately. All Radscores were constructed using the minimum λ. The logλ_min_ values of the four sequences were 0.00017 (T1WI, **Figure 1A**), 0.0051 (T2WI, **Figure 1B**), 0.00091(CET1WI, **Figure 1C**), and 0.0019 (DWI, **Figure 1D**). In total, 20 radiomic features were used to build Radscore_T1WI (**Figure 2A**), 17 for Radscore_T2WI (**Figure 2B**), 19 for Radscore_CET1WI (**Figure 2C**), and 19 for Radscore_DWI (**Figure 2D**). Additionally, the Radscore showed a significant correlation with height (P=0.00), weight (p=0.001), neutrophils (P=0.00), monocytes (P=0.004), serum albumin (P=0.004), SCC_Ag (P=0.00), HPV (P=0.00), tumor diameter on MRI (P=0.00), FIGO stage (P=0.00), depth of stromal invasion (P=0.001), and LVSI (P=0.00) as shown in **Supplementary Table 1**.

### 3.4 Diagnostic performance of Radscores

**Figure 4:**
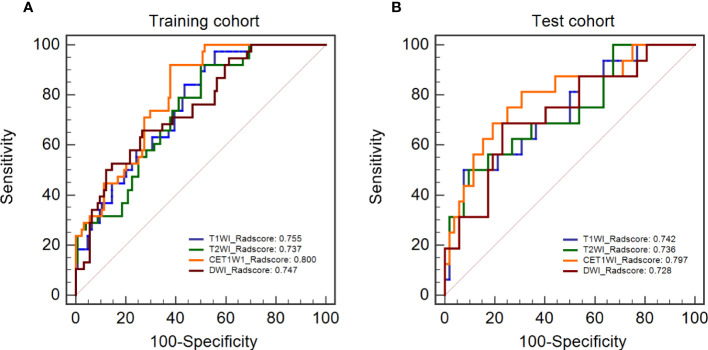
Diagnostic performance and DeLong test of Radscore in single-sequence models. **(A)** the diagnostic performance of the single-sequence Radscore in the training cohort. **(B)** the diagnostic performance of the single-sequence Radscore in the test cohort.

The Radscores per single sequence of patients were calculated according to the Radscore equations (**Equations 1**–**4**). All Radscores exhibited significant differences between NLM and LNM (P<0.05, **Figure 3**). The AUCs of Radscore_T1WI for predicting NLM and LNM were 0.755 (**Figure 4A**) and 0.742 in the training and test cohorts, respectively (**Figure 4B**). The AUCs of Radscore_T2WI for predicting NLM and LNM were 0.737 (**Figure 4A**) and 0.736 (**Figure 4B**) in the training and test cohorts, respectively. Additionally, the AUCs of Radscore_CET1WI for predicting NLM and LNM were 0.800 (**Figure 4A**) and 0.797 (**Figure 4B**) in the training and test cohorts, respectively. Further, the AUCs of Radscore_DWI for predicting NLM and LNM were 0.747 (**Figure 4A**) and 0.728 (**Figure 4B**) in the training and test cohorts, respectively.

**Equation 1:**
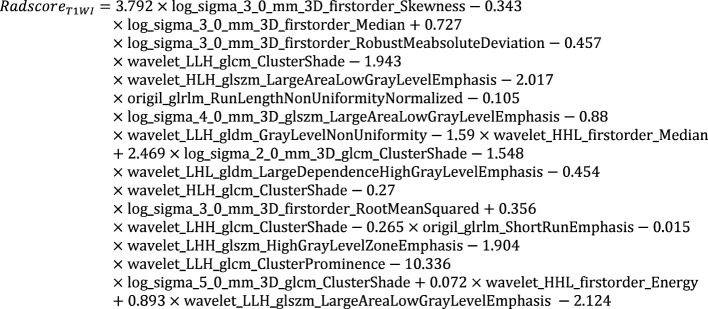


**Equation 2:**
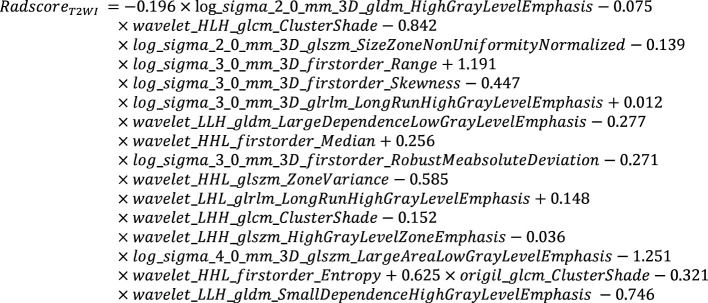


**Equation 3:**
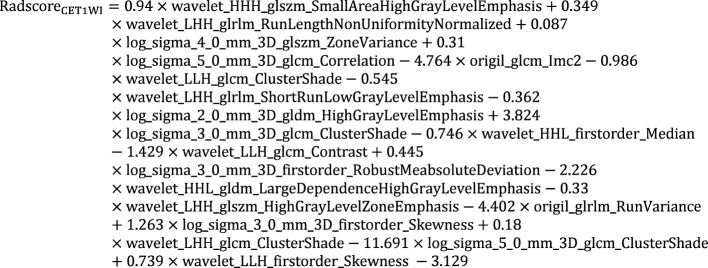


**Equation 4:**
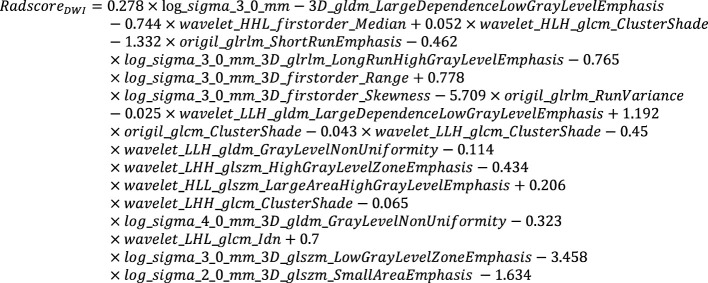


There were no statistically significant differences among the individual Radscores of the single-sequence model in either the training or test cohort (P>0.05, **Figures 4A, B** **)**. Therefore, Radscore_CET1WI was selected to be combined with other demographic and clinical variables based on the highest AUC to construct the Radscore_Clinical model (Rad_clin model). All the mean and standard deviation of features for the constructed Radscore_CET1WI are shown in **Table 3**.

**Table 3:** Mean and standard deviation of the features.

	NLM		LNM	
	mean	SD	mean	SD
wavelet_HHH_glszm_SmallAreaHighGrayLevelEmphasis	60.739	92.077	95.784	126.678
wavelet_LHH_glrlm_RunLengthNonUniformityNormalized	0.882	0.556	0.949	0.541
log_sigma_4_0_mm_3D_glszm_ZoneVariance	491.755	916.639	567.653	697.310
log_sigma_5_0_mm_3D_glcm_Correlation	0.709	0.518	0.680	0.216
origil_glcm_Imc2	4.126	10.328	1.932	3.266
wavelet_LLH_glcm_ClusterShade	17.786	293.155	54.743	213.037
wavelet_LHH_glrlm_ShortRunLowGrayLevelEmphasis	8.887E+07	5.571E+08	1.214E+07	3.217E+07
log_sigma_2_0_mm_3D_gldm_HighGrayLevelEmphasis	1.444E+09	1.282E+10	2.673E+07	1.068E+08
log_sigma_3_0_mm_3D_glcm_ClusterShade	-897.132	8932.455	-717.314	4720.700
wavelet_HHL_firstorder_Median	0.190	0.601	0.084	0.473
wavelet_LLH_glcm_Contrast	18.586	26.117	16.748	12.261
log_sigma_3_0_mm_3D_firstorder_RobustMeabsoluteDeviation	56.667	131.415	60.741	242.450
wavelet_HHL_gldm_LargeDependenceHighGrayLevelEmphasis	4.842E+03	8.278E+03	3.101E+03	3.625E+03
wavelet_LHH_glszm_HighGrayLevelZoneEmphasis	73.940	93.012	86.618	87.475
origil_glrlm_RunVariance	0.105	0.525	0.057	0.094
log_sigma_3_0_mm_3D_firstorder_Skewness	0.009	0.345	0.088	0.361
wavelet_LHH_glcm_ClusterShade	0.511	2.887	1.480	3.883
log_sigma_5_0_mm_3D_glcm_ClusterShade	1.048E+03	1.122E+04	-6.486E+02	4.953E+03
wavelet_LLH_firstorder_Skewness	0.522	0.901	0.699	0.749

NLM, non-lymphatic metastasis; LNM, lymph node metastasis; SD, standard deviation.

### 3.5 Radscore-clinical integrated model

**Figure 5:**
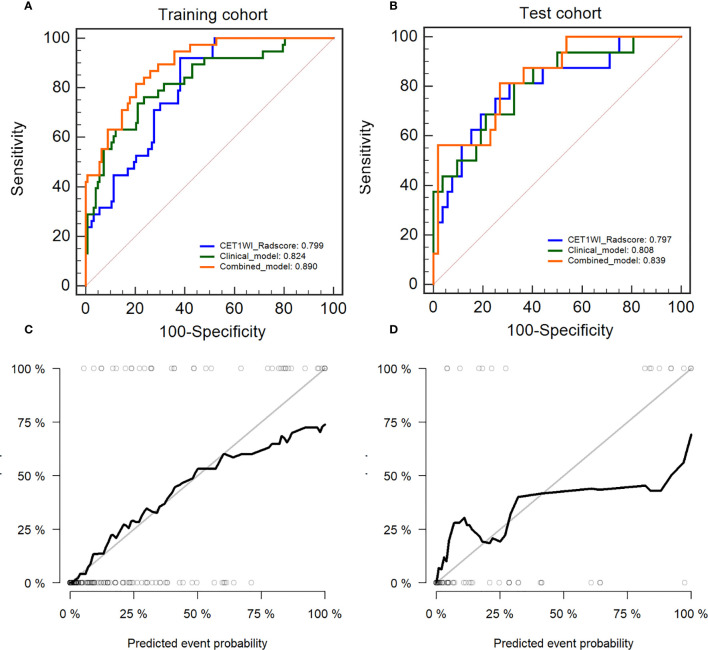
Diagnostic evaluation and test of the nomogram for the prediction of non-lymphatic metastasis (NLM) and lymphatic metastasis (LNM). The receiver operating characteristic (ROC) curves of Radscore_CET1WI, clinical parameters, and the nomogram in the **(A)** training cohort and **(B)** test cohort. Calibration curves for the nomogram in the **(C)** training cohort and **(D)** test cohort.

**Figure 6:**
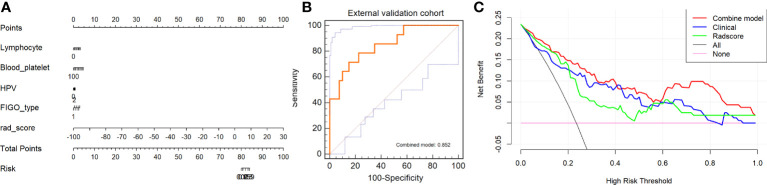
Model evaluation and test of nomogram for non-lymphatic metastasis (NLM) and lymphatic metastasis (LNM). **(A)** Nomogram based on clinical characteristics and Radscore_CET1WI. **(B)** ROC analysis of Rad_clin model in external validation cohort. **(C)** Decision curves for Rad_clin model, Radscore, and clinical parameters. The Y-axis shows the clinical benefit. The red line represents the nomogram (Rad_clin model), the blue line represents the clinical parameters, and the green line represents the Radscore. The X-axis shows the threshold of clinical risk. ROC, receiver operating characteristic; T1WI, T1-weighted imaging; T2WI, T2-weighted imaging; CET1WI, contrast-enhanced T1WI; DWI, diffusion-weighted imaging.

We constructed an integrated diagnosis model using a multiple logistic regression model in combination with clinical variables. After calculating the odds ratio (OR) of the clinical variables, lymphocyte (OR=1.91, 95% confidence interval [CI]: 1.18–3.35, P=0.015), blood platelet (OR=1.008, 95% CI: 1.00–1.01, P=0.001), SCC_Ag (OR=1.06, 95% CI: 1.00–1.12, P=0.021), CEA (OR=0.998, 95% CI: 0.986–1.00, P=0.663), HPV (OR=1.0006, 95% CI: 1.0001–1.0006, P=0.035), tumor diameter on MRI (OR=1.330, 95% CI: 1.01–1.77, P=0.044), and FIGO stage (OR=1.56, 95% CI: 1.29–1.91, P<0.0001) were maintained. Based on the minimum AIC, lymphocyte, blood platelet, HPV, and FIGO type were incorporated into the Rad_clin model constructed using Radscore_CET1WI. The Hosmer–Lemeshow test in the Rad_clin model revealed no significant differences in the goodness of fit for the training (P=0.594) or test cohort (P=0.748, **Equation 5**). We also calculated the discriminatory efficiency of the clinical parameters (diameter of the tumour_MRI), Radscore, and Rad_clin model using ROC analysis (**Table 4**; **Figures 5A–D**). The Rad_clin model yielded the largest AUC values in the training (0.890) and test (0.839) cohorts (**Table 4**). The DeLong test revealed no significant differences in diagnostic performance among the Radscore_CET1WI, Clinical, and Rad_clin models (P_radscore_ vs _Clinical_train_=0.662, P_radscore_ vs _Clinical_test_=0.904, P_Rad_clin_model_ vs _Clinical_train_=0.015, P_Rad_clin_model_ vs _clinical test_ =0.609, P_Rad_clin_model_ vs _radscore_train_=0.003, P_Rad_clin_model_ vs _radscore test_=0.229). The Rad_clin model was visualized using a nomogram (**Figure 6A**). The diagnostic performance of the Rad_clin model was 0.852 in the external validation cohort (**Figure 6B**).

**Equation 5:**
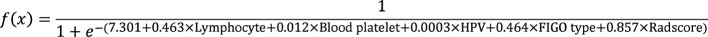


**Table 4:** Diagnostic performance of Radscore in the combined models.

	Cohort	AUC	95% CI		Accuracy	95% CI		Sensitivity	Specificity	PPV	NPV
			Lower	Upper		Lower	Upper				
Radscore_CET1WI	Training	0.800	0.731	0.873	0.691	0.614	0.761	0.921	0.621	0.427	0.962
	Test	0.797	0.670	0.934	0.676	0.552	0.784	0.812	0.635	0.406	0.917
Clinical	Training	0.824	0.741	0.902	0.765	0.692	0.828	0.763	0.766	0.500	0.913
	Test	0.808	0.683	0.931	0.691	0.567	0.798	0.687	0.692	0.407	0.878
Rad_clin model	Training	0.890	0.844	0.939	0.802	0.733	0.861	0.816	0.798	0.553	0.934
	Test	0.839	0.728	0.954	0.750	0.630	0.847	0.481	0.927	0.812	0.731

AUC, area under the curve; CI, confidence interval; NPV, negative predictive value; PPV, positive predictive value; CET1WI, contrast-enhanced T1-weighted imaging.

The results of the DCA analysis for the Radscore_CET1WI, Clinical, and Rad_clin models in the training and test cohorts are displayed in **Figure 6C**. The DCA analysis yielded a clinical risk threshold in the range of 0–0.8, which could benefit from the Rad_clin model.

## 4 Discussion

Researchers have increasingly focused on the capacity of radiomics to predict LNM before surgery in patients who have not received NACT, chemotherapy, or radiotherapy. However, given the advancements in medical technology, a single treatment plan is no longer used in clinics. For instance, a previous study noted that NACT can reduce the rate of LNM in some patients who cannot undergo direct surgical treatment (5). In the present study, we progressively optimized a single-sequence Radscore to obtain a superior diagnostic performance for distinguishing between NLM and LNM in patients with cervical cancer, which was observed with CET1WI. Joint analysis of a patient’s Radscore and clinical variables may be of great significance in the differential diagnosis of other diseases that are difficult to distinguish.

Radscore showed a significant correlation with height, weight, neutrophils, monocytes, serum albumin, SCC_Ag, HPV, tumor diameter on MRI, FIGO stage, depth of stromal invasion, and LVSI (**Supplementary Table 1**). However, the correlation values for these variables with Radscore were relatively low, suggesting a need for further investigation into the relationship between radiomics features and histological features or other clinical variables.

Additionally, we observed that the incidence of LNM in the NACT group was significantly lower than that in the surgery group (P=0.007) in this study. The rate of positive lymph nodes in patients receiving NACT plus surgery was lower than that in patients undergoing surgery alone (3, 8, 9). Several studies have demonstrated the diagnostic performance of radiomics for predicting LNM. However, in these studies, patients were excluded if they had received preoperative chemotherapy, radiation therapy, or NACT (11, 18, 19). Furthermore, these studies focused on the prediction of LNM before surgery because their purpose was to aid in treatment-related decision-making and evaluate the prognosis of patients with cervical cancer. Therefore, these models are only applicable to patients undergoing surgery alone. However, preoperative NACT has been confirmed to provide an increased survival benefit for patients with cervical cancer (4, 20). The NCCN suggested that patients with cervical cancer may receive NACT followed by RH (3). Therefore, we built a Radscore to predict LNM in patients who received NACT before surgery.

Increasing evidence indicates that radiomics analysis exhibits good performance in predicting tumors, especially when the Radscore is built using MRI (21). Nonetheless, the reproducibility and comparability of radiomics research can be improved *via* the normalization of imaging protocols, especially for mpMRI, which involves multiple parameters and sequences (9, 22). There are several ways to build a Radscore using MRI, such as the use of multiple sequences combined (21); however, we constructed four single-sequence-based Radscores using T1WI, T2WI, CET1WI, and DWI sequences separately. Radscore_CET1WI (AUCtrain=0.800 vs. AUCtest=0.797) was found to have the highest AUC value among all sequences, although there were no significant differences among the four sequences. We selected Radscore_CET1WI to be combined with other demographic and clinical variables to reduce the complexity of research operations and to construct the Rad_clin model. Li et al. also reported a Radscore based on CET1WI for predicting LVSI (23).

Since an increasing number of MRI-based radiomics studies have been conducted, it is necessary to consider the issue of wasting medical resources. According to the guidelines, mpMRI provides information regarding lesions and information on tumor cellularity and proliferation (24, 25). Contrast-enhanced MRI can also be used to non-invasively assess microcirculatory perfusion and vascular permeability of the tumor (26).

We constructed the Rad_clin model with superior diagnostic discrimination (AUCtrain=0.891, AUCtest=0.802) by combining Radscore_CET1WI with clinical features.

We found that a larger diameter on MRI (P=0.039) and higher FIGO stage (P<0.001) were common in patients with LNM in the training set and that a larger diameter on MRI and higher FIGO stage (P<0.001) were also common in patients with LNM in the test set. Lymphocytes, blood platelets, SCC_AG, and HPV were also higher in LNM than in NLM. Nonetheless, it is important to incorporate clinical variables in the radiomics analysis, where imaging is intended to complement and supplement rather than replace clinical decision-making. After calculating the variance inflation factor and AIC, lymphocyte, blood platelet, HPV, and FIGO type were included in the combined model. A recent study (27) reported that the tumor diameter on MRI may be a risk factor for predicting pathological LNM, which was not included in our Rad_clin model based on minimum AIC (15, 28). Previous studies have shown that blood platelet and lymphocyte are risk factors for LNM in patients with cervical cancer. Tumor-infiltrating CD4+ T cells and reversed CD4/CD8 ratios have been significantly associated with LNM in cervical cancer, indicating higher levels of lymphocytes and blood platelets, which aligns with our findings (29). Additionally, HPV and FIGO type have been identified as significant predictors of relapse and LNM in cervical cancer, especially correlating with poor prognosis, which is consistent with our results (30).

We used the external validation data to validate the diagnostic performance of the Radscore, clinical model, and Rad_clin model to reduce overfitting and improve repeatability. The Rad_clin model also showed a high AUC (0.852). It is worth noting that we did not normalize the images between the different centers. At the same time, the images from the different centers were scanned using different MRIs (21), which also yielded high AUC except for the clinical model. We believe that imaging over-processing should be avoided.

In advanced cervical cancer, radiochemotherapy, including the combination of external beam radiotherapy (EBRT) and brachytherapy (BRT) (31), can deliver high doses to the target lesion with good clinical outcomes and reduced adverse events. EBRT and BRT are considered the most important treatments for cervical cancer. Short-term treatment appears feasible; however, long-term outcomes should be advocated. Radiomics offers a non-invasive method for disease evaluation, making it a potential tool for predicting outcomes in patients undergoing EBRT and BRT.

There are some limitations to our study. The amount of data required is an ongoing topic in radiomics research. Our study included a small sample size; a larger sample size will make the model more stable and provide more reliable information. Most patients underwent MRI before NACT, and some patients received adjuvant chemotherapy after NACT. Furthermore, more cases across multiple centers should be included to verify the repeatability of this model. Lastly, the retrospective nature of our study allowed for the introduction of selection bias, limiting the potential generalizability of our results.

## 5 Conclusion

The Radscore provided by CET1WI, which included tumor diameter on MRI, may achieve high diagnostic performance in predicting LNM after NACT in patients with cervical cancer. Superior performance was observed using the Rad_clin model, which was constructed using the CET1WI-based Radscore and clinical/demographic variables.

## Data availability statement

The raw data supporting the conclusions of this article will be made available by the authors, without undue reservation.

## Ethics statement

The studies involving human participants were reviewed and approved by the Ethics Committee of the Third Affiliated Hospital of Kunming Medical University. The patients/participants provided their written informed consent to participate in this study. Written informed consent was obtained from the individual(s) for the publication of any potentially identifiable images or data included in this article.

## Author contributions

Guarantor of integrity of the entire study, CA, LaZ, LeZ & HH. Study concepts/study design or data acquisition of data analysis/interpretation: all authors. Manuscript drafting or manuscript revision for important intellectual content: all authors. Manuscript final version approval: all authors. Agrees to ensure any questions related to the work are appropriately resolved: all authors. Literature research: CA, LeZ, HH, SZ. Statistical analysis: WD and ZL. Manuscript editing: LeZ, HH, CA, SZ. All authors contributed to the article and approved the submitted version.
