# Supplementary material for: A nomogram-based optimized Radscore for preoperative prediction of lymph node metastasis in patients with cervical cancer after neoadjuvant chemotherapy
Source: Front Oncol. 2023 Aug 15;13:1117339. doi: 10.3389/fonc.2023.1117339 (PMC10466037; doi:10.3389/fonc.2023.1117339)
Supplement: Supplementary file 1 [file DataSheet_1.docx]

Supplementary Material

# Supplementary Figures and Tables

## Supplementary Figures

##
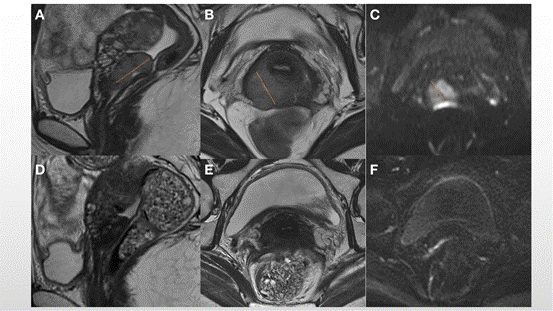


**Supplementary Figure 1.** Female who showed complete response after NACT. A, B & C: T2WI, T1WI, and DWI before NACT. D, E & F: T2WI, T1WI, and DWI after NACT. Red line showed the lesion.


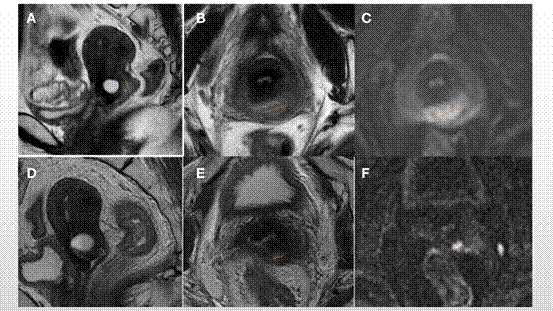


**Supplementary Figure 2.** Female who exhibited partial response after NACT. A, B & C: T2WI, T1WI, and DWI before NACT. D, E & F: T2WI, T1WI, and DWI after NACT. Red line shows the lesion.


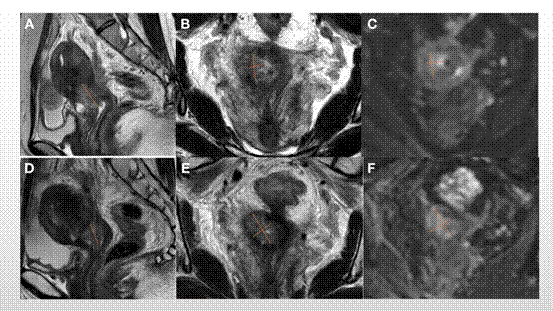


**Supplementary Figure 3.** Female who exhibited progressive disease after NACT. A, B & C: T2WI, T1WI, and DWI before NACT. D, E & F: T2WI, T1WI, and DWI after NACT. Red line shows the lesion.

## Supplementary Tables

|  | Pearson Correlation | P-value |
| --- | --- | --- |
| Age, years | 0.045 | 0.178 |
| Gestation number | -0.025 | 0.468 |
| Parturition number | -0.051 | 0.131 |
| Height, cm | 0.211 | 0* |
| Weight, kg | 0.111 | 0.001* |
| BMI | 0.004 | 0.903 |
| Neutrophils | 0.148 | 0* |
| Lymphocytes | 0.009 | 0.797 |
| Monocytes | 0.098 | 0.004* |
| Blood platelets | -0.06 | 0.075 |
| Hemoglobin | -0.056 | 0.099 |
| Serum albumin | -0.096 | 0.004* |
| SCC_Ag | 0.123 | 0* |
| CEA | 0.03 | 0.37 |
| HPV | 0.147 | 0* |
| Tumor diameter on MRI, cm | 0.26 | 0* |
| Diameter pathology, cm | -0.002 | 0.955 |
| FIGO stage | 0.217 | 0* |
| Histotypes, n | 0.034 | 0.317 |
| Depth of stromal invasion | -0.113 | 0.001* |
| Lymph vascular space invasion | -0.212 | 0* |

Supplementary Table 1. Correlation between Radscore and clinical variables

*significant difference
